# *Candida albicans* and *Candida glabrata* triosephosphate isomerase – a moonlighting protein that can be exposed on the candidal cell surface and bind to human extracellular matrix proteins

**DOI:** 10.1186/s12866-021-02235-w

**Published:** 2021-07-01

**Authors:** Dorota Satala, Grzegorz Satala, Marcin Zawrotniak, Andrzej Kozik

**Affiliations:** 1 Department of Analytical Biochemistry, Faculty of Biochemistry, Biophysics and Biotechnology, Jagiellonian University in Krakow, Kraków, Poland; 2 Department of Medicinal Chemistry, Maj Institute of Pharmacology, Polish Academy of Sciences, Kraków, Poland; 3 Department of Comparative Biochemistry and Bioanalytics, Faculty of Biochemistry, Biophysics and Biotechnology, Jagiellonian University in Krakow, Kraków, Poland; 4 Department of Analytical Biochemistry, Faculty of Biochemistry, Biophysics and Biotechnology, Jagiellonian University in Krakow, Gronostajowa 7, 30-384 Krakow, Poland

**Keywords:** non-albicans *Candida* species, triosephosphate isomerase, moonlighting proteins, extracellular matrix, vitronectin, fibronectin, collagen, laminin, elastin

## Abstract

**Background:**

Triosephosphate isomerase (Tpi1) is a glycolytic enzyme that has recently been reported also to be an atypical proteinaceous component of the *Candida* yeast cell wall. Similar to other known candidal “moonlighting proteins”, surface-exposed Tpi1 is likely to contribute to fungal adhesion during the colonization and infection of a human host. The aim of our present study was to directly prove the presence of Tpi1 on *C. albicans* and *C. glabrata* cells under various growth conditions and characterize the interactions of native Tpi1, isolated and purified from the candidal cell wall, with human extracellular matrix proteins.

**Results:**

Surface plasmon resonance measurements were used to determine the dissociation constants for the complexes of Tpi1 with host proteins and these values were found to fall within a relatively narrow range of 10^− 8^-10^− 7^ M. Using a chemical cross-linking method, two motifs of the Tpi1 molecule (aa 4–17 and aa 224–247) were identified to be directly involved in the interaction with vitronectin. A proposed structural model for Tpi1 confirmed that these interaction sites were at a considerable distance from the catalytic active site. Synthetic peptides with these sequences significantly inhibited Tpi1 binding to several extracellular matrix proteins suggesting that a common region on the surface of Tpi1 molecule is involved in the interactions with the host proteins.

**Conclusions:**

The current study provided structural insights into the interactions of human extracellular matrix proteins with Tpi1 that can occur at the cell surface of *Candida* yeasts and contribute to the host infection by these fungal pathogens.

**Supplementary Information:**

The online version contains supplementary material available at 10.1186/s12866-021-02235-w.

## Background

Secreted and cell surface-exposed proteins are among the most important tools used by pathogenic microorganisms to colonize a host organism, and to subsequently survive and multiply in the host tissues. These extracellular proteinaceous factors, best represented by the secreted hydrolytic enzymes and cell wall-anchored adhesins, are expressed in a tightly controlled response to environmental signals and not only allow microorganisms to firmly attach to host tissues, preventing them from being washed out, but also help these microbes to survive under unfavorable conditions within the infected host, such as limited nutrient availability, oxidative stress and the host immune response [1–4].

One of the mechanisms that has been relatively recently recognized to contribute to the pathogenicity of microorganisms is an *a priori* unexpected cell surface display of enzymes that are known to perform their basic, evolutionarily well-conserved functions in the cytosol, e.g. participate in fundamental metabolic pathways such as glycolysis, fermentation or protein synthesis. These “atypical” proteinaceous cell wall components belong to a wide subset of multifunctional proteins termed “moonlighting proteins” [5]. The intracellular/secreted moonlighting proteins of pathogenic bacteria and fungi play confirmed roles in their adhesion to host cells and tissues, the binding of many host proteins, and the evasion of host immune responses [6–9].

Multiple moonlighting proteins have been repeatedly identified on the cell surfaces of *Candida* spp. opportunistic yeast-like fungi, that commensally occur on the skin and mucous membranes of apparently healthy human individuals, sometimes causing relatively mild superficial infections. However, in patients with an impaired immune system, e.g. as a consequence of chemotherapy or organ transplantation, these yeasts can cause multi-organ and systemic candidiasis, which is often life-threatening [10]. For a number of decades, *C. albicans* has been the most common etiological factor in human candidiasis. More currently however, a progressive increase in the relative contribution to fungal infections in the human population has been reported for “non-albicans” *Candida* species, the most notably *C. glabrata*, *C. parapsilosis* and *C. tropicalis*. Of these, *C. glabrata*, which is closely related to non-pathogenic *Saccharomyces cerevisiae* yeast, is particularly interesting. Depending on the geographical region, *C. glabrata* ranks second or third as the most common causative agent for candidiasis, accounting for approximately 13–27 % of all cases [11]. Comparisons of a variety of *C. albicans* and *C. glabrata* virulence factors suggest that these microorganisms use somewhat different strategies to colonize and survive in the host organism. The main differences include an inability of *C. glabrata* to grow in hyphal forms and different type, profile and roles of extracellular aspartyl proteases produced by two species. *C. albicans* expresses a family of versatile secreted aspartyl proteases (Sap), also known as candidapepsins, released in remarkable amounts at the infection sites and capable of digesting numerous host proteins, whilst *C. glabrata* produces an unlike family of cell surface-bound yapsins, predominantly involved in cell wall maintenance [3, 12].

In our previous proteomic studies of candidal cell-wall proteins, we identified the presence of a new intracellular/surface moonlighting protein on the cell surface of “non-albicans” *Candida* species - triosephosphate isomerase (Tpi1) -  which remains poorly characterized at this location for any of the known prokaryotic or eukaryotic pathogens. The abundance of this enzyme at the candidal surface was found to vary depending on the prevailing environmental conditions [13, 14]. Moreover, Tpi1 was also identified in the *C. albicans* secretome [15]. The basic function of Tpi (E.C. 5.3.1.1) is the catalysis of a reversible interconversion between glyceraldehyde-3-phosphate (GAP) and dihydroxyacetone phosphate (DHAP) - an important metabolic branch point between glycolysis, gluconeogenesis and the pentose phosphate pathway [16]. We reported that Tpi1 isolated from the cell wall of *C. albicans* could bind components of the human plasma kinin-generating system including high-molecular weight kininogen, factor XII and prekallikrein [17]. Moreover, Tpi was shown to be upregulated during a biofilm formation by *C. albicans* alone or as part of a dual-species community with *Staphylococcus aureus* [18]. However, its level was reported in other studies to be downregulated after contact with macrophages [19, 20]. Interestingly, Tpi1 levels decrease in *C. albicans* yeast-like cells exposed to the antifungal agent, HWY-289, but not in the hyphal (filamentous) forms exposed to this drug [21]. It was also shown in prior reports that antibodies against Tpi1 were present in the sera of systemically infected mice and in the sera of patients with candidiasis, thus classifying Tpi1 as an immunoreactive protein [22–24].

Given the limited data on the presence and role of Tpi1 on the surface of candidal cells, we aimed in our current study to investigate Tpi1 exposure on the surface of *C. albicans* and *C. glabrata* under various growth conditions and evaluate its possible adhesin functions, manifested via the interaction with human extracellular matrix (ECM) proteins.

## Results

### Tpi1 is variably exposed on the surface of *Candida* spp. cells, which is dependent on the environmental conditions

**Fig. 1:**
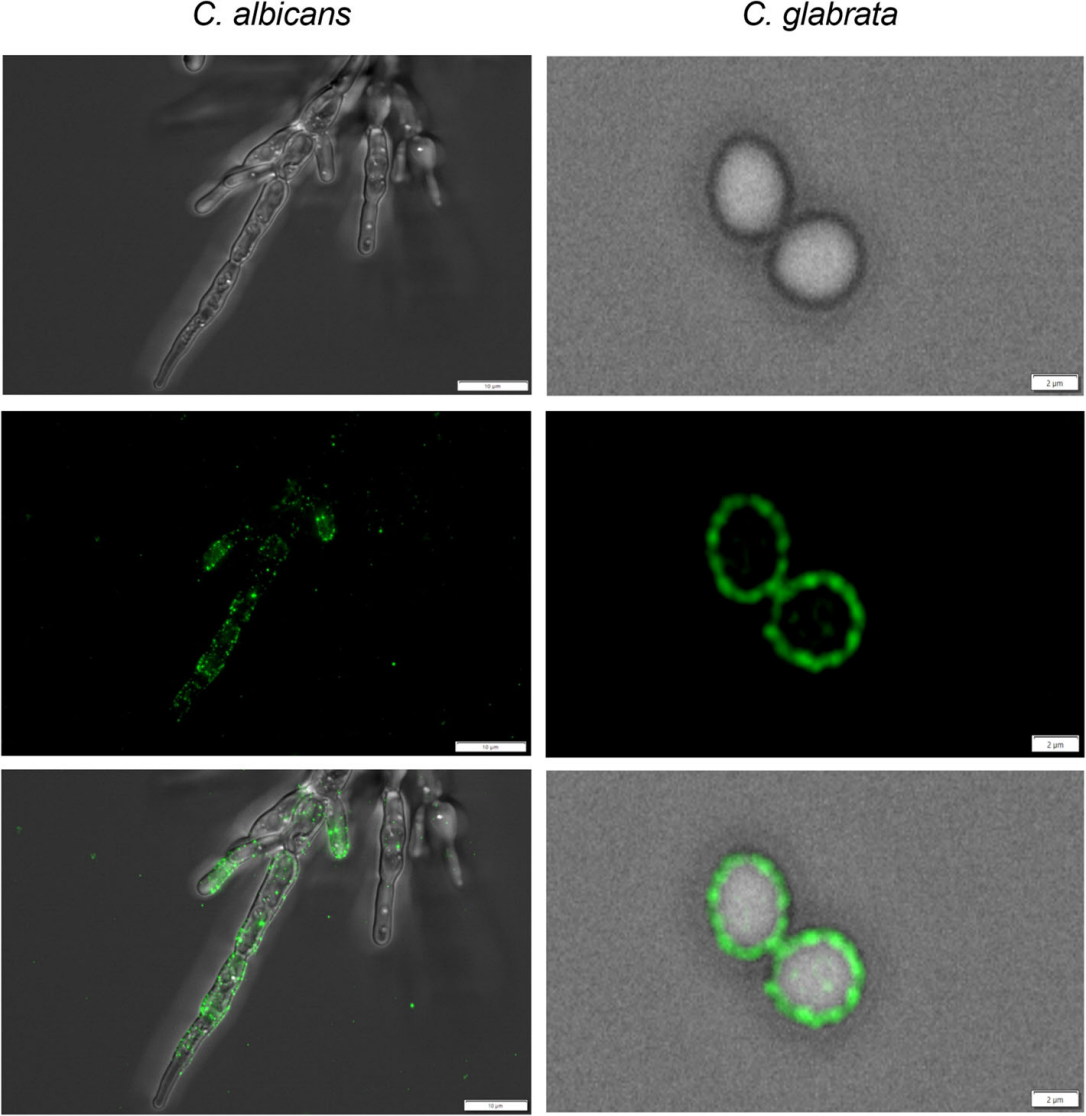
Identification of Tpi1 on the surface of *C. albicans* and *C. glabrata* cells grown in RPMI 1640 medium. *C. albicans* yeast-like cells, grown for 16 h in YPD medium at 30°C, were transferred to the wells of a glass bottom 96-well plate coated with poly-L-lysine (10^4^ cells per well) and incubated in RPMI 1640 medium at 37 °C overnight. *C. glabrata* yeast-like cells were grown overnight in RPMI 1640 medium in a glass flask and then transferred to Eppendorf tubes (3 x 10^7^ cells per tube). The presence of surface-exposed Tpi1 was detected using rabbit polyclonal anti-Tpi antibody (1 µg/mL) and an Alexa Fluor 488 fluorescently labeled anti-rabbit secondary antibody (1 μg/mL). The photos were taken using an Olympus IX73 microscope

Using antibodies against *S. cerevisiae* Tpi in conjunction with fluorescently-labelled secondary antibodies, we directly visualized the presence of Tpi1 on the surface of *C. albicans* and *C. glabrata* cells (Fig. 1). This observation was consistent with our previous identification of this enzyme in the cell wall proteomes of *C. glabrata*, *C. parapsilosis* and *C. tropicalis* [13, 14].

**Fig. 2:**
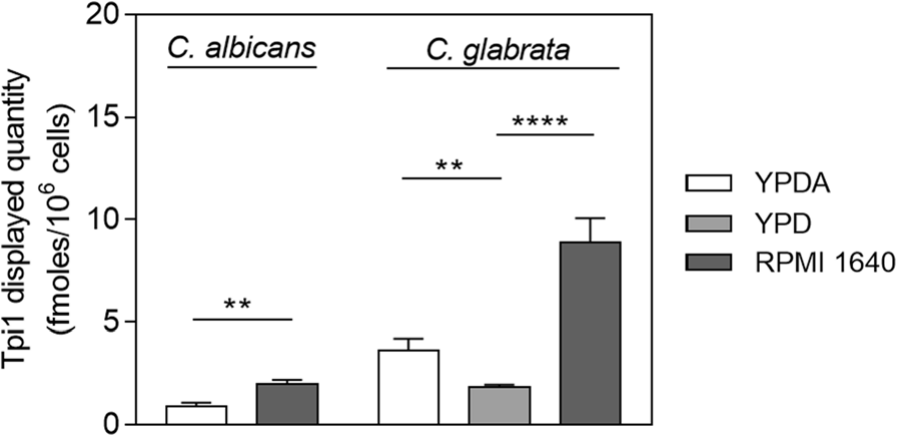
Identification of Tpi1 on the surface of *C. albicans* and *C. glabrata* cells grown under various conditions. For *C. albicans* cells, the experiment was performed in the wells of MaxiSorp microplates (1 x 10^6^ cells per well) whereas for C*. glabrata *cells, the experiment was carried out in Eppendorf tubes (3 x 10^7^cells per tube). The cells were grown under different culture conditions., i.e. YPD, YPDA or RPMI 1640, and were incubated with an anti-yeast Tpi1 antibody (1 µg/mL) followed by a HRP-conjugated secondary antibody. After washing off any unbound material, the level of bound antibodies was determined using TMB; however, in the case of *C. albicans* cultured in YPD medium the fluorescence signals were not measurable (as shown in Figure S2 in the Supplementary material). The obtained results were normalized for the same number of cells (1 x 10^6^) and for the different cross-reactivities of the applied anti-*S. cerevisiae* Tpi1 antibody with *C. glabrata* and *C. albicans *(based on the calibration presented in Figure S1A in the Supplementary material). Bars represent the mean values from three determinations, with standard deviations. Statistical significance levels were determined by one-way ANOVA; **p<0.01 and ****p<0.0001

It is known that the composition of the candidal proteome depends on the culture conditions, which can cause fungal cells to change their morphological forms between yeast-like cells and hyphae [14, 25, 26]. Accordingly, in our current study using anti-Tpi1 antibodies, we evaluated Tpi1 exposition on the surface of live *C. glabrata* and *C. albicans* cells grown under different culture media, namely YPD medium in which cells of both species exist as yeast-like forms, YPDA medium characterized by a reduced amount of nitrogen due to the presence of animal peptone and RPMI 1640 medium, which is typically used to induce hyphal growth of *C. albicans* (Fig. 2). The displayed amount of Tpi1 on the cell surface was calculated based on the obtained calibration curves for the purified enzymes in which cross-reactivity of anti-*S. cerevisiae* Tpi1 antibody with the *C. glabrata* and *C. albicans* enzyme was analyzed (Fig. S1A in the Supplementary material). *C. albicans* adopted a unicellular yeast form only when grown in rich, complete YPD medium. In the defined RPMI 1640 medium and YPDA medium *C. albicans* produced hyphal forms which are considered to be most invasive type of these fungal cells [27, 28].

**Fig. 3:**
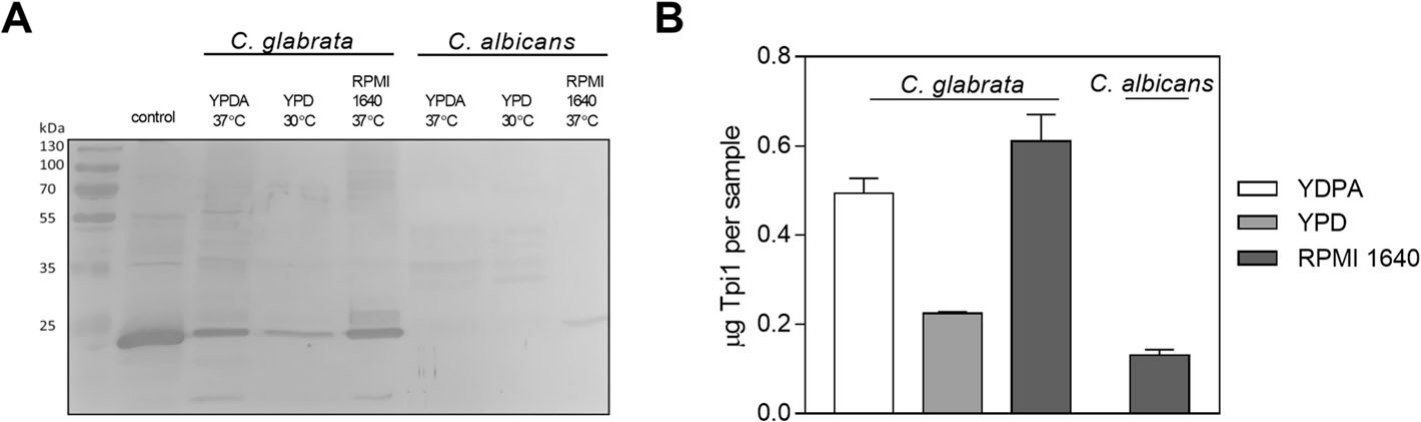
Western-blotting identification of Tpi1 in whole mixtures of cell wall proteins from *C. albicans* and *C. glabrata*. (**A**) Equal amounts of total proteins (20 µL at a concentration of 0.5 mg/mL) isolated under different culture conditions, i.e. YPD, YPDA and RPMI 1640 medium, were resolved by SDS-PAGE in the Laemmli system under reducing conditions. Thereafter, the protein bands were transferred to a PVDF membrane and probed with a primary anti-Tpi1 antibody and an alkaline phosphatase-labelled secondary antibody. Protein bands were visualized using a BCIP/NBT system. The uncropped scan of the blot is presented. 1 µg of *S. cerevisiae* Tpi was used as a control. (**B**) The Tpi1 amount within the mixture of *Candida* spp. cell wall proteins was determined by densitometric analysis of the obtained blots using ImageJ software. The raw results were corrected for the slightly different reactivities of anti-*S. cerevisiae* Tpi1 antibody with the purified Tpi protein from *C. glabrata* and *C. albicans* (based on the calibration presented in Figure S1B in the Supplementary materials). Bars represent the mean values with standard deviations from two independent experiments

On the basis of a semi-quantitative test, we detected Tpi1 on the surfaces of *C. glabrata* cells grown under various conditions, suggesting that this is a general phenomenon for this species. In *C. albicans* however, a preferential accumulation of Tpi1 on the hyphal surface was noted. This phenomenon may be related to the change in the total amount of intracellular Tpi1 as it was shown in a prior study that the amount of *C. albicans* Tpi1 increased by more than two-fold in the stationary phase compared with the exponential phase [29]. Similarly, a preferential presentation on the surface of hyphal forms was recently noted for the major *C. albicans* moonlighting protein - enolase [30]. Additionally, we performed western blotting analysis in our current study of mixtures of candidal cell wall proteins extracted from the fungal cells with β-1,6-glucanase (Fig. 3). For both species, we again observed the highest Tpi1 exposition on the surface of fungal cells after culturing in RPMI 1640 medium, suggesting a role of this protein in host infection. In *C. albicans* cultured in YPDA and YPD medium, Tpi1 could not be detected at the cell surface. In conclusion, based on our findings with three different experimental approaches, it was clearly evident from our current analyses that Tpi1 is present on the surface of *Candida* spp., and that the amount of exposed protein varies for each species, depending on the environmental conditions.

### Interaction of Tpi1 on the surface of *C. albicans* and *C. glabrata* cells with human extracellular matrix proteins

**Fig. 4:**
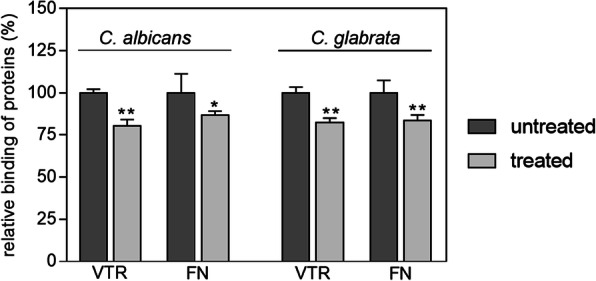
Use of anti-Tpi1 antibodies to displace labeled VTR and FN from binding to *C. albicans* and *C. glabrata *cells. Binding experiments were performed for *C. albicans* and C*. glabrata* in the wells of MaxiSorp microplates (1 x 10^6^ cells per well) or in Eppendorf tubes (3 x 10^7^ cells per tube), respectively. The candidal cells grown in RPMI 1640 medium were pre-incubated with anti-*S. cerevisiae* Tpi1 antibodies (1 µg/mL) followed by 50 µL of biotin-labeled human VTR or FN proteins at a final concentration of 250 nM. This was followed in all cases by an incubation for 1.5 h at 37°C. After washing off unbound protein, the amounts of bound VTR or FN were determined using an SA-HR/TMB system. The level of binding of the biotinylated human protein to the *Candida *spp. cells without antibodies was considered to be 100%. Bars represent the mean values from three determinations, with standard deviations. Statistical significance levels were determined using a one-way ANOVA test against the signal from the control sample containing the biotinylated proteins with no antibody pre-incubation; *p<0.05 and **p<0.01

To confirm the involvement of candidal surface-exposed Tpi1 in the binding of host ECM components, an antibody-based displacement test was performed. Two ECM proteins, vitronectin (VTR) and fibronectin (FN), were analyzed for their interaction with *Candida* spp. cells cultured in RPMI 1640 medium. This medium was chosen because the amounts of Tpi1 on the surface of both species was found to be highest under these culturing conditions. In the microplate assay, biotinylated VTR and FN showed binding to the surface of *C. albicans* and *C. glabrata* cells that decreased by about 20 % after cell pre-treatment with anti-Tpi antibodies, compared with the level of total binding determined for human proteins in the absence of these antibodies (Fig. 4). This experiment provided a good foundation for a working hypothesis that Tpi1 exposed on the surface of *Candida* spp. could be a potential partner for the interaction of this microbe with host extracellular matrix proteins.

To further develop the above hypothesis and characterize the direct interaction between fungal proteins and human ECM proteins, Tpi1 exposed at the surface of *C. albicans* and *C. glabrata* cells was purified using the strategy previously described that allowed for effective isolation of this protein from the whole mixture of cell wall proteins by means of ion-exchange chromatography and gel filtration [17]. The purity of obtained proteins was evaluated by sodium dodecyl sulphate-polyacrylamide gel electrophoresis (SDS-PAGE) (Fig. S3 in the Supplementary material) and their final identification was confirmed by liquid chromatography-coupled tandem mass spectrometry (LC-MS/MS). Tpi1 was found to be enzymatically active and not to contain attached sugar moieties, thus excluding the possibility of this type post-translational modification during the transition from the cytosol to the cell surface.

**Fig. 5:**
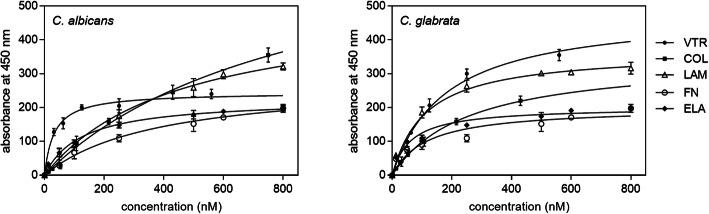
Binding of purified *C. albicans* and *C. glabrata *Tpi1 to human ECM proteins, analyzed using a microplate-based protein-binding assay. Microplate wells were coated with human proteins (3 pmoles per well) in an overnight incubation at 4 ˚C. Solutions of biotin-labeled Tpi1 in 50 μL PBS at a concentration range of 10–800 nM were then added to the walls and the microplates were incubated for 1.5 h at 37 °C. After washing off the unbound protein, the amounts of bound biotinylated Tpi1 were determined using the SA-HRP/TMB system. The values obtained for the control samples, represented by the wells without human proteins, were subtracted from the total binding. Data points represent the mean values of three determinations, with standard deviations

The interactions of purified Tpi1 with several different ECM proteins were next directly demonstrated using a microplate-based protein-binding assay that allowed us to estimate and compare overall binding levels (Fig. 5). It was found that each of the ECM proteins tested interacted with the purified candidal Tpi1, and the apparent dissociation constant (*K*_*D*_) was estimated to be in an order of 10^− 7^ M for most of the studied protein-protein pairs. The strongest binding (*K*_*D*_ of approximately 10^− 8^ M) was determined for the *C. albicans* Tpi1-VTR pair while the weakest binding (*K*_*D*_ of an order of 10^− 6^ M) was determined for the *C. glabrata* Tpi1-collagen (COL) pair.

**Fig. 6:**
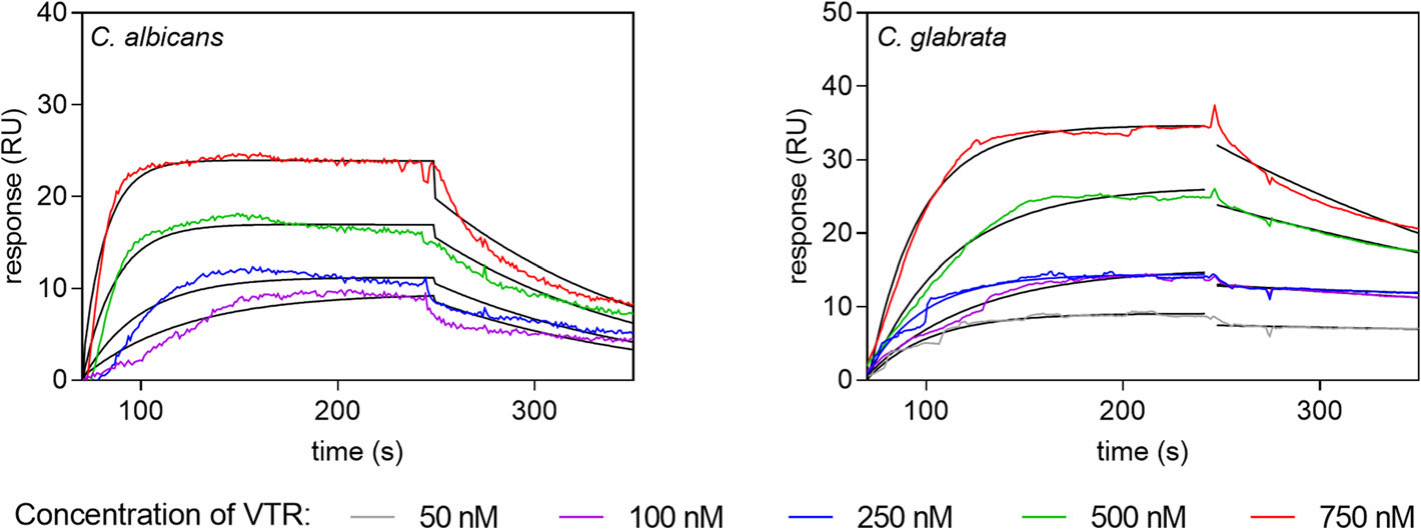
SPR sensograms for the interactions between candidal Tpi1 and human VTR. VTR solutions in a buffer containing 10 mM HEPES, 150 mM NaCl and 0.005 % (w/v) P20 surfactant, pH 7.4, at concentrations in a range of 50-750 nM, were injected onto a CM5 chip containing immobilized Tpi1 (150 RU) at a flow rate of 30 μL/min for 180 seconds. A 1:1 Langmuir binding model was fitted to the experimental data (black lines)

A more rigorous method based on surface plasmon resonance (SPR) measurements was used to determine kinetic and thermodynamic parameters of the protein-protein interactions studied, including the association rate constant (*ka*), the dissociation rate constant (*kd*) and the equilibrium dissociation constant *K*_*D*_ (*K*_*D*_*= kd*/*ka*). In these experiments, candidal Tpi1 was immobilized on the CM5 chip of the Biacore 3000 system, and human protein solutions were injected over the chip surface. This approach seemed to better model the *in vivo* situation in which Tpi1 is embedded in the fungal cell wall structure and possibly interacts with soluble human proteinaceous targets. Example sensograms obtained for the Tpi1–VTR pairs are presented in Fig. 6, and all other sensograms are shown in Figure S4 in the Supplementary material. Good, global or local fits to sensograms were obtained using the Langmuir 1:1 binding model. The obtained dissociation constant (*K*_*D*_) values were in the range of 10^− 8^ – 10^− 7^ M, indicating a strong interaction of Tpi1 with human proteins. The strongest binding (*K*_*D*_ of an order of 10^− 8^ M) was measured for the *C. albicans* Tpi1-VTR and *C. glabrata* Tpi1-elasin (ELA) interacting pairs (Table 1).

**Table 1:** Kinetic and thermodynamic parameters of fungal Tpi1 binding by ECM proteins, determined by SPR measurements

ECM protein	*k*_*a*_ (1/Ms)	*k*_*d*_ (1/s)	*K*_*D*_ (M)
*C. albicans* Tpi1			
VTR	1.02 x 10^5^ ± 3.16 x 10^3^	8.77 x 10^-3^ ± 7.40 x 10^-4^	8.60 x 10^-8^ ± 5.28 x 10^-9^
FN	3.22 x 10^4^ ± 3.99 x 10^3^	1.05 x 10^-2^ ± 5.08 x 10^-4^	3.26 x 10^-7^ ± 2.73 x 10^-8^
LAM	4.42 x 10^4^ ± 4.09 x 10^3^	3.27 x 10^-2^ ± 1.68 x 10^-3^	7.39 x 10^-7^ ± 7.99 x 10^-8^
COL	1.82 x 10^4^ ± 2.48 x 10^3^	1.99 x 10^-3^ ± 3.10 x 10^-4^	1.09 x 10^-7^ ± 9.16 x 10^-9^
ELA	4.65 x 10^4^ ± 7.79 x 10^3^	3.82 x 10^-2^ ± 1.56 x 10^-3^	8.21 x 10^-7^ ± 8.04 x 10^-8^
*C. glabrata* Tpi1			
VTR	1.84 x 10^4^ ± 9.84 x 10^2^	3.10 x 10^-3^ ± 1.04 x 10^-3^	1.68 x 10^-7^ ± 4.13 x 10^-9^
FN	6.48 x 10^4^ ± 5.23 x 10^3^	4.73 x 10^-2^ ± 1.36 x 10^-3^	7.30 x 10^-7^ ± 7.24 x 10^-9^
LAM	1.34 x 10^4^ ± 4.05 x 10^3^	3.39 x 10^-3^ ± 2.62 x 10^-4^	2.53 x 10^-7^ ± 3.18 x 10^-8^
COL	1.44 x 10^4^ ± 1.90 x 10^3^	3.08 x 10^-3^ ± 2.91 x 10^-4^	2.14 x 10^-7^ ± 2.86 x 10^-8^
ELA	9.51 x 10^4^ ± 4,83 x 10^3^	1.91 x 10^-3^ ± 9.04 x 10^-5^	2.01 x 10^-8^ ± 1.97 x 10^-8^

The binding parameters for interactions between fungal proteins and human ECM proteins were determined after global fitting of the data with a 1:1 Langmuir binding model. All parameters are presented with standard errors

### Chemical cross-linking and molecular modeling to map the moonlighting interactions of enzymatically active Tpi1 with human vitronectin

To obtain structural insights into the moonlighting interaction of candidal Tpi1 with host ECM proteins, we chose VTR as a model ligand due to its relatively small size and strongest affinity for Tpi1, as determined by SPR (see above). We first checked whether the formation of the Tpi1–VTR complex affected the Tpi1 enzymatic activity. Since we did not observe any significant inactivation of Tpi1 after incubation with human proteins at a 1:1 molar ratio (Fig. S5 in the Supplementary material), we speculated that the human protein binding site on fungal Tpi1 is at a considerable distance from the active site of this enzyme.

To identify the *C. albicans* Tpi1 sequence motifs that are directly involved in the interaction with VTR, chemical cross-linking was performed using a heterobifunctional photoreactive reagent, sulfosuccinimidyl 2-([4,4′-azipentanamido]ethyl)-1,3′-dithiopropionate (sulfo-SDAD). After attachment in the dark to amino residues on the human protein, the reagent was allowed to link to the nearest fragment of Tpi1 under UV light. After cleavage of the disulfide bridge in the sulfo-SDAD cross-link, the proteins were digested with trypsin and the resulting peptides were analyzed by LC-MS/MS. Using a Mass Matrix server, two peptides were finally identified – _4_QFFVGGNFKANGTK_17_ and _224_ANVDGFLVGGASLKPEFVDIIKSR_247_ – that had a mass shift consistent with the mass of the sulfo-SDAD fragment (for details of Mass Matrix analysis, see Table S1 in the Supplementary material).

**Fig. 7:**
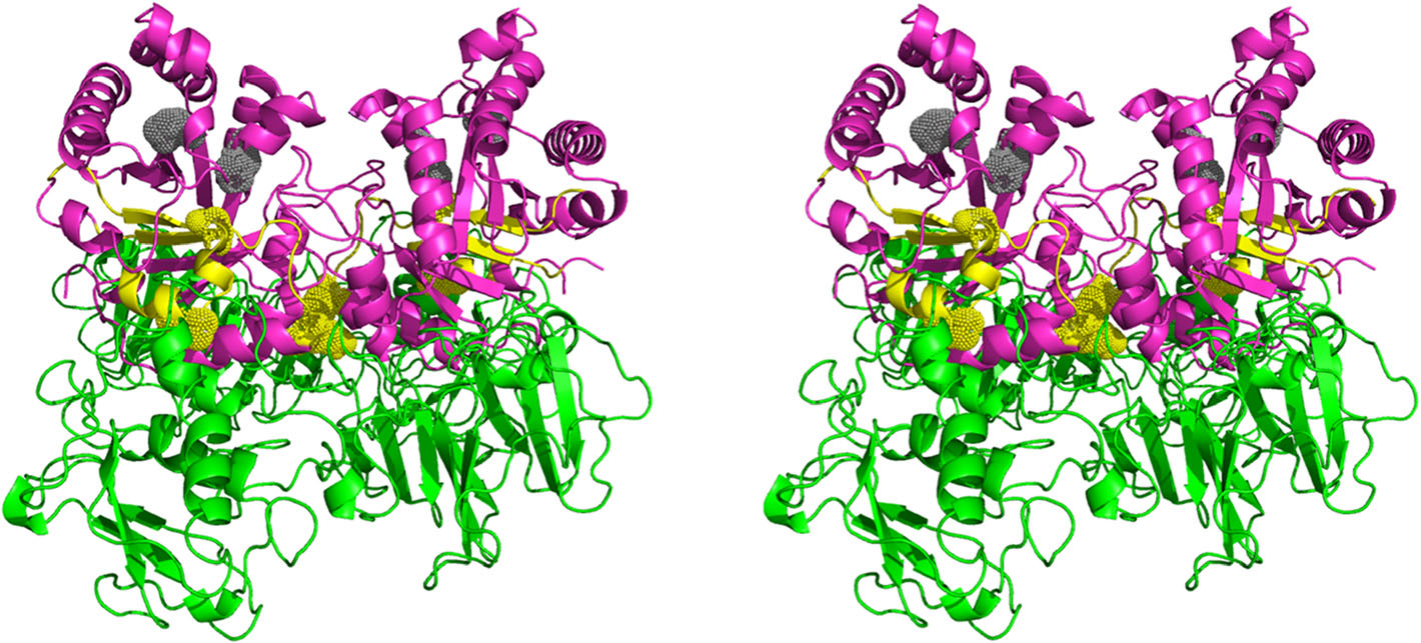
Wall-eyed stereo view of a model for the interaction between *C. albicans* Tpi1 and VTR. The indicated dimer of *C. albicans* Tpi1 (purple), with an active site denoted in gray, was obtained by homology modeling with Modeller software, based on the* S. cerevisiae* Tpi structure (66 % identity; PDB, 3 ypi). Tpi1 amino acids, marked in yellow, are directly involved in the interaction with VTR (green), as found by chemical cross-linking. An additional movie file shows this interaction in more detail (see Supplementary material Video S1)


After mapping the Tpi1 fragments involved in the interaction with VTR, a structural model of this interaction was proposed using the ClusPro 2.0: protein-protein docking software. A dimeric structure of *C. albicans* Tpi1 obtained by homology modelling based on the known crystal structure of *S. cerevisiae* Tpi (PDB: 3ypi), and a VTR structure obtained using Schrödinger (LLC), were applied to the docking prediction. The obtained model (Fig. 7 and Video S1 in the Supplementary material) confirmed that the interaction of Tpi1 with host proteins should not affect its original enzymatic function because the identified Tpi1 peptides (marked in yellow in the schematic) were located at a considerable distance from the Tpi1 active site, which remains unoccupied (marked in grey).

**Additional file 2: Video S1.** Video presentation of the interaction between *C. albicans* Tpi1 and VTR.

### Common sequence motifs on Tpi1 molecule interact with different human extracellular matrix proteins

**Fig. 8:**
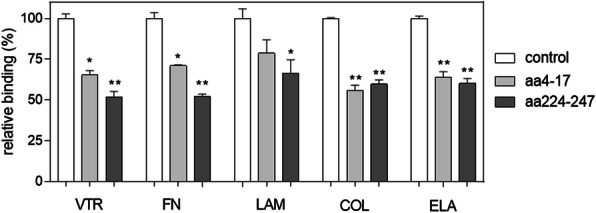
Displacement of *C. albicans *Tpi1 from ECM proteins by peptides matching Tpi1 fragments. Samples containing 25 µL of biotinylated Tpi1 (300 nM) and 25 µL of the specified peptide solution (30 µg/mL) were added to the microplate wells containing 3 pmoles of immobilized ECM proteins and the microplates were incubated for 1.5 h at 37 °C. After washing off unbound protein, the amounts of bound Tpi1 were determined using the SA-HR/TMB system. The level of binding of the biotinylated Tpi1 without a competitor added was considered as 100 %. Bars on graph represent the mean values of three determinations, with standard deviations. Statistical significance levels were determined using a one-way ANOVA test against the control sample; *p<0.05 and **p<0.01

Synthetic peptides with sequences that match those of putative VTR-binding motifs of Tpi1 molecule (aa4-17 and aa224-247) were used as inhibitors of the interactions between biotinylated Tpi1 and microplate-immobilized ECM proteins (Fig. 8). These two peptides were shown to displace, respectively, 35 and 50 % of Tpi1 from binding to VTR, thus confirming the findings from cross-linking experiments and molecular modelling. Comparable inhibitory effects of both peptides were observed for Tpi1 binding to FN, laminin (LAM), COL and ELA, with some quantitative differences however, such as the weakest inhibition of Tpi1-LAM binding or more equivalent inhibitory strength of aa4-17 and aa224-247 peptides in the Tpi1-interactions with COL and ELA than with VTR and FN. These data suggest that the detected sequences on Tpi1 molecule represent new internal motifs involved in binding of different host ECM proteins.

## Discussion

Tpi1, hitherto poorly characterized as a moonlighting protein from *Candida* species, has been detected on the cell surface of a few microbial pathogens such as a bacterium *Streptococcus anginosus* [31], a protist *Trichomonas vaginalis* [32] and a dimorphic fungus *Paracoccidioides brasiliensis* [33]. Moreover, the surface presence of Tpi has also been reported for multicellular parasites such as *Taenia solium* and *Schistosoma mansoni*, responsible for cysticercosis and schistosomiasis, respectively. Tpi exposed on the surface of their larvae was suggested to be a potential vaccine and drug target [34–36]. Interestingly, for *Staphylococcus aureus*, Tpi was suggested to be a potential adhesive molecule involved in the interaction between this bacterium and a fungal pathogen *Cryptococcus neoformans* via recognition of the skeleton of *C. neoformans* mannooligosaccharides [37].

In our present analyses, we aimed to better characterize the moonlighting adhesin function of Tpi1 on the cell surface of two medically important *Candida* species, *C. albicans* and *C. glabrata*. First, we unequivocally confirmed the fungal cell surface exposition of Tpi1, using three independent approaches and employing anti-*S. cerevisiae* Tpi1 antibodies. The relative amounts of the surface form of Tpi1 were found to strongly depend on the environmental conditions, with the highest stimulation of surface Tpi1 exposition in the cells grown in RPMI 1640 medium, which is known to induce significant changes in the candidal proteome composition and also a morphological transition to hyphae in *C. albicans* [14, 25, 26]. These results are similar to the prior observations in another fungal pathogen, *P. brasiliensis*, in which Tpi1 surface expression increases when it adopts a pathogenic yeast-like morphology [33, 38].

The mechanisms of non-conventional secretion and cell-surface retardation of various fungal moonlighting proteins, including Tpi1, have not been satisfactorily characterized. This enzyme was identified within the cargo of extracellular vesicles, released by *P. brasiliensis* [38, 39] and *C. albicans* [40, 41]. However, Tpi1 was not found in these extracellular structures in *C. glabrata* [41], possibly reflecting differences in the virulence mechanisms of these two *Candida* species [12]. Another mechanism, based on a readsorption of cytosolic proteins released to the extracellular environment onto the surface of functional cells, has recently been identified in our laboratory for *C. albicans* enolase, a major candidal moonlighting protein, and suggested in the experiments to occur via binding to a major adhesin, agglutinin-like sequence protein 3 (Als3) [30]. This has not yet been investigated however for candidal Tpi1 or any other moonlighting protein.

One of the major manifestations of the adhesive properties of pathogen cell surface proteins is their ability to interact with host ECM proteins [6, 9, 42–45]. In addition to providing physical support for the cells that it surrounds, the ECM actively participates in the process of tissue and organ growth and proliferation, providing them with a stable environment by regulating the number of growth factors and receptors, and maintaining an adequate level of hydration and pH of the microenvironment (for reviews see: [46, 47]). Multiple functions of the ECM rely not only on its complex structure, but also a dynamic reconstruction in response to environmental stimuli, such as an applied force or an injury. Furthermore, the ECM can also affect cellular activity and metabolic processes by activating intracellular signaling pathways, as well as impact the cell shape and migration capacity by affecting the functioning of the cytoskeleton [48]. The main components of the ECM are the glycosaminoglycans and fibrillar proteins responsible for its mechanical function, primarily including COL and ELA. Other important proteinaceous ECM components are FN, a multi-domain glycoprotein with a molecular mass of about 440 kDa, LAM, a family of multifunctional glycoproteins with molecular masses in a range of 400–900 kDa, and VTR, a multi-domain glycoprotein with a molecular mass of about 75 kDa [49, 50]. Unlike other ECM components, VTR does not have a structural function and its tasks include binding to other proteins such as cytokines or receptors present on the surfaces of cells [51].

The significance of the interactions between host ECM proteins and *Candida* spp. cells in the development of fungal infections has been documented for *C. albicans*. The ability of *C. albicans* strains to bind to proteins such as FN, LAM and COL was shown to correlate with relative strain pathogenicity [52]. Although in the case of *C. albicans* several molecules capable of interacting with the ECM proteins were indicated, such as the classic adhesins - Als1, Als3 and Als5, and moonlighting proteins such as alcohol dehydrogenase, glyceraldehyde-3-phosphate dehydrogenase and enolase [9, 53–56], the structural mechanisms underlying these interactions remain poorly understood. Bacterial Tpi1 has been reported to be involved in the binding of LAM, FN and plasminogen [31, 32], whilst recombinant *P. brasiliensis* Tpi interacts with pneumocytes by binding to LAM and FN [38, 39]. Here, we characterized the binding of candidal Tpi1 to selected human ECM proteins, considering that these interactions are important for the attachment of *Candida* yeast to host tissues and, consequently, for host infection. An anti-Tpi1 antibody decreased the adsorption of ECM proteins (FN and VTR) to live candidal cells by 20 %, suggesting this level of Tpi1 contribution to the overall binding of ECM proteins by the fungal cells. It should be noted also however that many other candidal cell surface proteins in addition to Tpi1, including both true adhesins and multiple moonlighting proteins, collectively contribute to the total capacity of ECM protein binding by candidal cells [9].

We directly demonstrate from our present results that purified Tpi1 exhibits binding activity towards ECM proteins. We did so using a microplate-based protein assay that allowed us to roughly estimate the overall effectiveness of the protein-protein interactions of interest. Quantitatively, the kinetic parameters for these interactions were determined by SPR measurements. Currently, such physicochemical characteristics of fungal cell wall protein binding to host ECM proteins have been presented in only a few reports. To date, FN- and LAM-binding to the N-terminal fragment of Als1 [57, 58], FN- and VTR-binding to *C. albicans* enolase [59], and interactions of FN with *C. glabrata* Epa6, and with the N-terminal fragment *C. glabrata* Epa1 [60, 61], have been characterized using SPR. In our current work, the dissociation constants (*K*_*D*_) for individual Tpi1-ECM protein pairs were determined to be in a range of 10^− 8^ – 10^− 7^ M, suggesting relatively strong interactions. In our previous study on the interactions of several *C. albicans* cell-surface proteins with components of the human plasma kinin-forming system [17], Tpi1 demonstrated the strongest binding to high molecular mass kininogen and factor XII, with a *K*_*D*_ < 10^− 8^ M. For other candidal proteins, most interestingly even for the true GPI-anchored adhesin Als3, the binding was markedly weaker, with several fold higher *K*_*D*_ values.

It is hypothesized that additional extracellular activities (i.e. moonlighting properties) of proteins that perform evolutionally conserved functions in intracellular metabolism may have evolved through adjustments of previously unused surfaces of these molecules as alternative binding sites for non-canonical ligands, as long as the core enzymatic function was unaffected by these structural adaptations [5, 6]. To add to this hypothesis in relation to the candidal Tpi1-human ECM protein interactions examined in our present study, we aimed to gain deeper insights into the structure of a representative complex, i.e. between *C. albicans* Tpi1 and VTR. To our knowledge, we have here presented the first structural analysis of the moonlighting interactions of Tpi1 in any organism. With the use of chemical cross-linking, combined with mass spectrometry analysis, we identified two Tpi1 sequence motifs putatively located within the contact area between the two proteins in the complex i.e. _4_QFFVGGNFKANGTK_17_ and _224_ANVDGFLVGGASLKPEFVDIIKSR_247_. Interestingly, the corresponding sequences in the *C. glabrata* Tpi1 are identical, suggesting similar arrangements in the interactions between the human VTR protein and Tpi1 from the latter species. Based on our results from these experiments, we developed a model of the complex between *C. albicans* Tpi1 and VTR using molecular modelling software. One interesting feature of this model was that the putative VTR-binding site on Tpi1 is well separated from the enzymatic activity center. This is consistent with the results of our direct assay of Tpi1 enzymatic activity, which remained unchanged in the presence of a VTR excess, additionally suggesting that the classic catalytic site in Tpi1 is unaffected by any possible conformational adjustments to the moonlighting (VTR binding) site upon VTR binding. Similar features were recently reported for complexes of candidal enolase with ECM proteins or human plasminogen [59]. This lack of a cooperation between sites seems therefore to be a general rule for evolutionally conserved intracellular enzymes that additionally moonlight at cell surfaces via the binding of non-canonical ligands.

In the last step of our analysis, we verified the role of the detected Tpi1 fragments responsible for VTR binding as potential internal motifs involved in the interactions with other host proteins. In the displacement test performed, the binding of Tpi1 to other ECM components – FN, LAM, COL and ELA, was competitively inhibited in the presence of synthetic peptides matching Tpi1 sequence fragments of aa4–17 and aa224–247. These data suggest that the identified motifs represent common regions of Tpi1 molecule that are recognized by a range of different host proteins.

## Conclusions

Our current findings indicate that the candidal glycolytic enzyme, Tpi1, is exposed on the surface of *C. albicans* and *C. glabrata* and exhibits a previously unrecognized function as an adhesin capable of interacting with numerous host proteins such as the main extracellular matrix components. A better understanding of the molecular functions and mechanisms of this moonlighting protein, and its involvement in the adhesion processes, may contribute to a development of new methods for identifying the etiological factors for candidiasis by using it as a potential diagnostic marker.

## Methods

### Yeast strains and growth conditions

The *C. albicans* strain ATCC® 10,231™ isolated from a man with bronchomycosis and *C. glabrata* strain CBS138 (ATCC® 2001™) isolated from human feces were purchased from American Type Culture Collection (Manassas, VA) and grown according to previously published protocols [13, 30]. Yeasts were cultured in YPD medium containing 1 % yeast extract, 2 % soybean peptone and 2 % glucose at 30˚C for 16 h with shaking at 170 rpm (MaxQ 4000, Thermo Fisher Scientific, Waltham, MA), in YPD buffered medium with a lowered content of animal-derived peptone (YPDA), pH 7.0 (0.1 % yeast extract, 0.2 % peptone from animal proteins, 2 % glucose and 10 mM NaH_2_PO_4_) at 37 °C for 48 h, or in a defined medium, RPMI 1640, pH 7.4 at 37 °C for 48 h, to induce the hyphal forms of *C. albicans* or to modify the surface-protein exposition by *C. glabrata* yeast-like cells.

## Commercial proteins and peptides

Human VTR was purchased from R&D Systems (Minneapolis, MN), human FN and ELA from Sigma-Aldrich Co. (St. Louis, MO), human LAM and COL from Merck Millipore (Burlington, MA), rabbit polyclonal antibody to *S*. *cerevisiae* Tpi from LSBio (Seattle, WA), goat anti-rabbit alkaline phosphatase-labelled antibody with 5-bromo-4-chloro-3-indolyl-phosphate/blue tetrazolium substrate (SIGMA*FAST™* BCIP®/NBT) used as a substrate from Sigma-Aldrich Co., Alexa Fluor 488-labelled anti-rabbit secondary antibodies from Abcam (Cambridge, UK), peroxidase-conjugated goat anti-rabbit antibody from Santa Cruz Biotechnology (Dallas, TX), β-1,6-glucanase from Takara Bio Inc. (Otsu, Shiga, Japan), trypsin from Promega (Madison, WI), bovine serum albumin (BSA) from BioShop Canada Inc. (Burlington, ON, Canada), and horseradish peroxidase-conjugated streptavidin solution (SA-HRP) from MP Biomedicals (Solon, OH). Peptides with amino acid sequences of QFFVGGNFKANGTK and ANVDGFLVGGASLKPEFVDIIKSR that match Tpi1 aa4–17 and aa224–247 fragments were custom-synthesized by GenScript (Piscataway Township, NJ).

### Antibody-based detection of Tpi1 on the surface of candidal cells

#### Microscopic observations of Tpi1 on the surface of living fungal cells

Surface-exposed Tpi1 was detected as previously described [30] with minor modifications. *C. albicans* yeast-like cells grown for 16 h in YPD medium at 30 °C were transferred to Eppendorf tubes (3 × 10^7^ cells per tube) or transferred to the wells of a glass-bottomed 96-well plate (CellVis, Mountain View, CA) coated with poly-L-lysine (10^4^ cells per well) and incubated in RPMI 1640 medium or YPDA medium at 37 °C overnight. After each step, the fungal cells were washed three times with 200 µL of phosphate-buffered saline (PBS). Prior to Tpi1 detection, yeast cells were incubated with 3 % BSA for 1 h at 37 °C to reduce any non-specific binding. The immunodetection of surface-exposed Tpi1 was performed with the addition of 50 µL of rabbit anti-Tpi antibodies (1 µg/mL, LSBio) in PBS for 1 h at 37 °C, and, subsequently, with Alexa Fluor 488 fluorescently labelled anti-rabbit secondary antibodies (1 µg/mL, Abcam) in PBS for 1 h. For *C. glabrata*, yeast-like cells grown for 16 h in YPD medium at 30 °C, or grown overnight in RPMI 1640 medium or YPDA medium in a glass flask were transferred to Eppendorf tubes (3 × 10^7^ cells per tube), with analogous treatment. In the last step, cells were placed into the wells of a 96-well plate (CellVis). Imaging was performed using an Olympus IX73 microscope (Olympus, Tokyo, Japan), and image analysis was performed using CellSense software (Olympus).

#### Tpi1 display on the fungal cell surface under various growth conditions

The method described previously [30] was followed with some modifications. An antibody-based test for the presence of Tpi1 on *C. glabrata* cells and yeast form of *C. albicans* cultured in YPD medium was performed in Eppendorf tubes in which 3 × 10^7^ cells cultured in YPD, YPDA or RPMI 1640 media were placed. After each step, the cells were washed three times with 200 µL PBS, pH 7.4, additionally containing 1 % BSA and were centrifuged between washes (3 min, 3000 g). After the addition of 50 µL of rabbit polyclonal anti-Tpi1 antibodies (1 µg/mL) in PBS, the tubes were incubated at 37 °C for 1 h. Next, 50 µL of peroxidase-conjugated secondary antibody was added and the tube was incubated again at 37 °C for 1 h. After washing, the cells were transferred to new Eppendorf tubes and then treated with SA-HRP, followed by the addition of 3,3’,5,5 ‘-tetramethylbenzidine (TMB) (Sigma-Aldrich) as a HRP substrate, as described previously [62]. *C. glabrata* cells to which only secondary antibodies were added provided a control for non-specific binding.

The Tpi1 localization assay for the hyphal form of *C. albicans* was performed as above with some modifications. Briefly, *C. albicans* cells were plated on MaxiSorp 96-well microtiter plates (Nunc, Roskilde, Denmark) (1 × 10^6^ cells per well) and cultured for 3 h at 37 °C in YPDA or RPMI 1640 medium. Before adding antibodies, the unoccupied well surface was blocked with 200 µL 3 % BSA in PBS for 1 h at 37 °C.

Calibrating experiments were also carried out on MaxiSorp 96-well microtiter plates with the use of anti-*S. cerevisiae* Tpi1 antibody and peroxidase-conjugated secondary antibodies, with the same incubation times and washing method, except that the wells of the microplates were covered with Tpi1 in amounts in a range of 0.5–4.5 fmoles (50 µL of protein solution in PBS) during an overnight incubation at 4 °C. The amounts of Tpi1 presented on yeast cells’ surface were calculated from the absorbance readings after SA-HRP/TMB detection.

#### Western blotting analysis of Tpi1 from mixtures of isolated cell wall proteins

Cell wall-associated proteins were isolated using previously published protocols [45]. Briefly, fungal cells (YPD-, YPDA- or RPMI 1640-cultured, 1 g wet weight) were placed in 1 mL of McIlvaine buffer containing a mixture of 0.1 M citric acid and 0.2 M disodium phosphate, pH 6.0, with 0.5 M sodium tartrate as an osmotic stabilizer and treated with 2 U of β-1,6-glucanase for 24 h at 37 °C. After extraction and centrifugation, the supernatants were collected and dialyzed against PBS, pH 7.4, at 4 °C for 48 h. Cell membrane integrity was tested by staining with Trypan Blue (Sigma-Aldrich).

The obtained protein mixtures were analyzed SDS-PAGE in the Laemmli system, followed by their transfer onto polyvinylidene fluoride (PVDF) membranes (Immobilon, Millipore) for 40 min at 250 mA in 10 mM CAPS buffer, pH 11.0 with 10 % methanol [63]. The membranes were blocked overnight at 4 °C in TTBS buffer (1 mM Tris, 15 mM NaCl, 0.05 % Tween 20, pH 7.6) containing 5 % non-fat milk. After rinsing with TTBS buffer, the membranes were probed with primary antibodies against Tpi1 and a secondary alkaline phosphatase-labelled antibody. The protein bands were visualized using BCIP/NBT and quantified by densitometry using ImageJ software [64]. In addition, parallel western blotting analysis was performed for the purified *C. albicans* Tpi1 and *C. glabrata* Tpi1 to evaluate the cross-reactivities of the anti-*S. cerevisiae* Tpi antibodies with the candidal enzymes (Fig. S1B in the Supplementary material).

### Purification of surface-exposed forms of *C. albicans* and *C. glabrata* Tpi1

After culturing *C. albicans* and *C. glabrata* cells in RPMI 1640 medium, a mixture of surface-exposed proteins from yeast cells was isolated via the β-1,6-glucanase enzyme using the procedure described above for western blotting. For the purification of *C. albicans* Tpi1, a previously described method was used [17] that included ion-exchange chromatography on a Mono Q column (GE Healthcare/Pharmacia, Uppsala, Sweden) and gel filtration on a Superdex-200 column (Amersham Bioscience, UK). For the purification of *C. glabrata* Tpi1, a similar procedure with some modifications was applied [60]. Briefly, in the first stage, ion-exchange chromatography on a Resource Q column (Pharmacia Biotech, Uppsala, Sweden) was used. The Tpi1-containing fractions were then injected onto a TSK G 3000 SW column (Tosoh Bioscience, PA). The purity of obtained proteins was assessed by SDS-PAGE and their identity was confirmed by LC-MS/MS, following previously described protocols [13]. Using a glycosylation assay kit (Pierce ™ Glycoprotein Staining Kit, Thermo Fisher Scientific), Tpi1 was found not to contain attached sugar moieties (data not shown). In addition, the activity of the purified Tpi1 was analyzed using a colorimetric assay kit (Abcam, Cambridge, UK) following the manufacturer’s instructions. In this assay, Tpi1 converts DHAP into GAP, which then reacts with the enzyme mix and developer to form a colored product with a strong absorbance at 450 nm. One unit (U) of Tpi is the amount of enzyme that generates 1.0 µmol of NADH per minute at pH 7.4 and 37 °C. The specific enzymatic activities of purified Tpi1 obtained in the current study from two *Candida* species were comparable with each other, in an order of 30 units per mg protein.

### Labelling of purified Tpi1, VTR and FN

For protein biotinylation, a 1 mg/100 µL solution of N-hydroxysuccinimide ester of biotin (Sigma-Aldrich) in dimethylformamide was added to protein solutions that had been pre-dialyzed against 0.1 M bicarbonate buffer, pH 8.3, maintaining a ratio of 10 µg NHS-biotin per 50 µg protein. After a 4 h incubation at 4 °C, the mixture was dialyzed against PBS buffer, pH 7.4 for 48 h at 4 ˚C. For Tpi1 labelling with fluorescein, a solution (1 mg/100 µL) of fluorescein N-hydroxysuccinimide ester (NHS-fluorescein, Thermo Fisher Scientific) in dimethylformamide was added to Tpi1 solution pre-dialyzed against a 0.1 M bicarbonate buffer, pH 8.3, maintaining a 15-fold molar excess of the label. The mixture was then incubated for 2 h at 4 ˚C in the dark. The excess of the labelling reagent was removed by dialysis to PBS buffer, pH 7.4 for 48 h at 4 ˚C.

### Use of anti-Tpi antibody to displace human VTR or FN from binding to *C. albicans* or *C. glabrata* cells

*C. albicans* cells were grown for 16 h in YPD medium at 30 °C. The yeast-like cells were then transferred to the wells (10^6^ cells per well) of MaxiSorp 96-well microtiter plates (Nunc, Roskilde, Denmark) and incubated in RPMI 1640 medium for 3 h at 37 °C. After each step of the following assay, the cells were washed three times with 200 µL PBS buffer containing 1 % BSA and the unoccupied well surface was blocked with 3 % BSA for 1 h at 37 °C. After washing, 50 µL of anti-*S. cerevisiae* Tpi1 antibodies (1 µg/mL) were added to the hyphae for a 1 h incubation at 37 °C with gentle mixing. Solutions of biotinylated human proteins (250 nM in a total volume of 50 µL per well) were then added, and the microplates were incubated for 1.5 h at 37 °C. The amount of bound biotinylated human protein was determined using the streptavidin-horseradish peroxidase/tetramethylbenzidine system (SA-HRP/TMB). Cells that were incubated with PBS instead of anti-Tpi1 antibodies represented 100 % relative binding. An analogous experiment was carried out for *C. glabrata* cells after culturing in RPMI 1640 medium, with some minor modifications. Briefly, the yeast-like cells were transferred into Eppendorf tubes (3 × 10^7^ cells per tube). After each step, the cells were washed three times with 200 µL PBS containing 1 % BSA and were centrifuged between washes (3 min, 3000 g). Before detection of the binding level, cells were transferred to new Eppendorf tubes.

## Analysis of Tpi1 binding to microplate-immobilized extracellular matrix proteins

Individual ECM proteins (3 pmoles per well) were immobilized in the wells of a MaxiSorp 96-well microplate (Sarstedt, Nümbrecht, Germany) through an overnight incubation at 4 °C. After each step of the following assay, the wells were washed three times with 200 µL PBS containing 1 % BSA. The unoccupied surface in each well was blocked with 3 % BSA in PBS for 3 h at 37 °C. Solutions of the pre-labelled fungal Tpi1, prepared in PBS at increasing concentrations (10–800 nM), were added followed by an incubation for 1.5 h at 37 °C. After washing out the unbound proteins, the binding levels were detected with the SA-HRP/TMB system.

### Characterization of the interactions between fungal Tpi1 and human extracellular matrix proteins using SPR measurements

The basic kinetic and thermodynamic characterization of the interactions between ECM proteins and purified Tpi1 was performed via SPR measurements in a BIACORE 3000 system (GE Healthcare), following the procedures described previously [17]. Briefly, after activation of the carboxyl groups present on the CM5 sensor chip (GE Healthcare) with a mixture of 50 mM 1-ethyl-3- (3-dimethylaminopropyl) carbodiimide (EDC) and 200 mM N-hydroxysuccinimide (NHS), Tpi1 was immobilized to a level of ca. 150 response units (RUs). The immobilization procedure was carried out at 25 °C, with a flow rate of 10 µL/min, in acetate buffer at pH 4.5. The binding level of the ECM proteins was analyzed in a running buffer containing 10 mM HEPES, 150 mM NaCl and 0.005 % (w/v) P20 surfactant, pH 7.4. Human protein solutions at variable concentrations (a range of 50–750 nM) were injected over the chip at a flow rate of 20 µL/min for the association and dissociation time of 3 min. The chip surface between the binding cycles was regenerated by injection of 1 M NaCl for 30 s at a flow of 30 µL/min. BIAevaluation 4.1.1 software (GE Healthcare) was used to estimate the binding parameters, based on a global or local fit with a simple Langmuir model, i.e. a 1:1 interaction.

### Chemical cross-linking mapping of *C. albicans* Tpi1 fragments involved in the interactions with VTR

VTR (20 µg, 100 µL), pre-dialyzed against PBS buffer, pH 7.4, was incubated in the dark at 4 °C for 2 h with 0.5 mM sulfo-SDAD (Thermo Fisher Scientific). After that time, 50 mM Tris was added to the mixture and the sample was incubated on ice for 15 min to stop the reaction. After removing excess cross-linking reagent by overnight dialysis, Tpi1 (20 µg in 100 µL PBS) was added to the sample. The mixture was then incubated at 37 °C for 1 h in the dark, with gentle shaking. The sample was next placed on ice and exposed to UV radiation (365 nm) for 15 min (6 W, Vilber Lourmat), followed by an overnight dialysis against 25 mM ammonium bicarbonate buffer, pH 8.0, at 4 °C. Subsequently, the VTR-Tpi1 adducts were reductively cleaved with 50 mM dithiothreitol at 60 °C for 60 min, followed by an alkylation with 55 mM iodoacetamide at room temperature for 45 min in the dark. Trypsin (3 µL of a 10 ng/µL solution in 25 mM NH_4_HCO_3_) was then added and the sample was incubated overnight at 37 °C. The reaction was stopped by a 5-min incubation with 2 M HCl. Samples were then centrifuged for 15 min at 12,000 rpm, dried in a Speed-Vac (Martin Christ, Osterode am Harz, Germany) and frozen until further use.

### Peptide identification by LC-MS/MS

For LC-MS/MS analysis, tryptic peptides were re-dissolved in 10 % acetonitrile with 0.1 % formic acid and analyzed with a HCT ultra ion-trap mass spectrometer. This instrument was equipped with an electrospray ionization ion source and an electron transfer dissociation II fragmentation module (Bruker, Bremen, Germany), and coupled to an ultra-high-performance liquid chromatograph Dionex Ultimate 3000 system (Thermo Fisher Scientific). Peptides were separated on a 100 mm × 2.1 mm Aeris 3.6 μm PEPTIDE XB-C18 column (Phenomenex, Torrance, CA), with a gradient of 10–60 % of 0.1 % formic acid in 80 % acetonitrile for 60 min, at a flow rate of 0.2 µL/min. The raw data were pre-processed using Data Analysis 4.0 software (Bruker), and the generated files, in Mascot Generic format (.mgf), were analyzed using a Mass Matrix PC version 4.3 server [65]. The following search parameters were applied: enzyme - trypsin; missed cleavages − 3; peptide length − 4 to 50 residues; mass tolerances − 0.6 Da; fragmentation – CID; and score thresholds of 4 for the pp and pp2, and 1.3 for pptag. The chemical formula of the sulfo-SDAD between protein and sulphur was specified as C_7_OSNH_10_ with a monoisotopic mass of 156.048 Da. The windows version of the program is available at http://www.massmatrix.net/download/.

### Molecular modelling of the interaction between *C. albicans* Tpi1 and human VTR

Molecular modelling of *C. albicans* Tpi1 (uniport ID: Q9P940) was carried out using Modeller software v9.14 [66] using the crystal structure of *S. cerevisiae* Tpi (PDB ID: 3YPI; the Research Collaboratory for Structural Bioinformatics Protein Data Bank, http://rcsb.org) as a template. The VTR structure (uniport ID: P04004, vitronectin V65 subunit) was obtained with Prime software (Schrödinger Release 2019-1, Schrödinger LLC, New York, NY) as previously described [59]. Protein Preparation Wizard (Schrödinger LLC) was used to prepare the final protein structure. The model of interaction between human VTR and fungal Tpi1 was proposed using ClusPro 2.0: protein-protein docking software (Boston University), a server version of which is available at https://cluspro.bu.edu. After docking the rigid body, two computational steps were performed to group 1,000 structures with the lowest energy based on root-mean-square deviation (RMSD) and removed steric collisions by minimizing the energy [67, 68]. The protein complexes obtained with this software were analyzed by comparing the distances between experimentally selected Tpi1 amino acid residues and the docked VTR using PyMOL Molecular Graphics System software (version 1.7.2.1; Schrödinger, LLC).

### Analysis of the displacement of biotinylated *C. albicans *Tpi1 from microplate-immobilized ECM proteins by peptide competitors

Individual ECM proteins (3 pmoles per well) were immobilized in the wells of a MaxiSorp 96-well microplate (Sarstedt, Nümbrecht, Germany) by overnight incubation at 4 °C. After each step of the following assay, the wells were washed three times with 200 µL PBS containing 1 % BSA. The unoccupied surface in each well was blocked with 3 % BSA in PBS by overnight incubation at 4 °C. The solutions, containing biotinylated Tpi1 at a final concentration of 300 nM (25 µL in PBS) and a peptide competitor at a final concentration of 30 µg/ml (25 µL in PBS), were added to the wells together, and the plate was incubated for 1.5 h at 37 °C. After washing out the unbound proteins, the binding level was detected with the SA-HRP/TMB system.

### Statistical analysis

Statistical analysis was performed using GraphPad Prism 8 (GraphPad Software, San Diego, CA). Statistical significance was assessed by one-way ANOVA followed by a Tukey’s test for multiple comparisons.

## Supplementary Information

**Additional file 1: Table S1.** List of peptide matches obtained with the Mass Matrix server. **Figure S1.** The analysis of cross-reactivities of anti-*S. cerevisiae* Tpi1 antibody to *C. albicans* and *C. glabrata* Tpi1. **Figure S2.** Identification of Tpi1 on the surface of *C. albicans* and *C. glabrata* cells, grown in YPD and YPDA medium. **Figure S3.** SDS-PAGE analysis of purified Tpi1 from *C. albicans* and *C. glabrata*. **Figure S4.** SPR sensograms for the interactions of *C. albicans* and *C. glabrata* Tpi1 with human ECM proteins: FN, LAM, COL and ELA. **Figure S5.** Relative enzymatic activity of *C. albicans* and *C. glabrata* Tpi1 after interaction with human ECM proteins.

## Data Availability

All data generated or analysed during this study are included in this published article and its supplementary information files. The row data analysed during the current study are available from the corresponding author on reasonable request.

## Abbreviations

BSA: bovine serum albumin.
BCIP/NBT: 5-bromo-4-chloro-3-indolyl-phosphate/blue tetrazolium substrate.
COL: collagen.
ECM: extracellular matrix.
ELA: elastin.
FN: fibronectin.
LAM: laminin.
LC-MS/MS: liquid chromatography-coupled tandem mass spectrometry.
SA-HRP/TMB: streptavidin-horseradish peroxidase/tetramethylbenzidine.
SDS-PAGE: sodium dodecyl sulphate polyacrylamide gel electrophoresis.
sulfo-SDAD: sulfosuccinimidyl 2-([4,40-azipentanamido]ethyl)-1,30-dithiopropionate.
SPR: surface plasmon resonance.
Tpi1: triosephosphate isomerase.
VTR: vitronectin.

## References

[1] Kline KA, Falker S, Dahlberg S, Normark S, Henriques-Normark B (2009). Bacterial adhesins in host-microbe interactions. Cell Host Microbe.

[2] Willaert RG (2018). Adhesins of yeasts: protein structure and interactions. J Fungi.

[3] Rapala-Kozik M, Bochenska O, Zajac D, Karkowska-Kuleta J, Gogol M, Zawrotniak M, Kozik A (2018). Extracellular proteinases of *Candida* species pathogenic yeasts. Mol Oral Microbiol.

[4] Nobile CJ, Mitchell AP (2006). Genetics and genomics of *Candida albicans* biofilm formation. Cell Microbiol.

[5] Jeffery CJ (2005). Mass spectrometry and the search for moonlighting proteins. Mass Spectrom Rev.

[6] Karkowska-Kuleta J, Kozik A (2014). Moonlighting proteins as virulence factors of pathogenic fungi, parasitic protozoa and multicellular parasites. Mol Oral Microbiol.

[7] Jeffery CJ (2018). Intracellular proteins moonlighting as bacterial adhesion factors. AIMS Microbiol.

[8] Wang G, Xia Y, Cui J, Gu Z, Song Y, Chen YQ (2014). The roles of moonlighting proteins in bacteria. Curr Issues Mol Biol.

[9] Satala D, Karkowska-Kuleta J, Zelazna A, Rapala-Kozik M, Kozik A (2020). Moonlighting Proteins at the Candidal Cell Surface. Microorganisms.

[10] Horn DL, Neofytos D, Anaissie EJ, Fishman JA, Steinbach WJ, Olyaei AJ (2009). Epidemiology and outcomes of candidemia in 2019 patients: data from the prospective antifungal therapy alliance registry. Clin Infect Dis.

[11] Lamoth F, Lockhart SR, Berkow EL, Calandra T (2018). Changes in the epidemiological landscape of invasive candidiasis. J Antimicrob Chemother.

[12] Brunke S, Hube B (2013). Two unlike cousins: *Candida albicans* and *C. glabrata* infection strategies. Cell Microbiol.

[13] Karkowska-Kuleta J, Zajac D, Bochenska O, Kozik A (2015). Surfaceome of pathogenic yeasts, *Candida parapsilosis* and *Candida tropicalis*, revealed with the use of cell surface shaving method and shotgun proteomic approach. Acta Biochim Pol.

[14] Karkowska-Kuleta J, Satala D, Bochenska O, Kozik A (2019). Moonlighting proteins are variably exposed at the cell surfaces of *Candida glabrata*, *Candida parapsilosis* and *Candida tropicalis* under certain growth conditions. BMC Microbiol.

[15] Gil-Bona A, Monteoliva L, Gil C (2015). Global proteomic profiling of the secretome of *Candida albicans* ecm33 cell wall mutant reveals the involvement of Ecm33 in Sap2 secretion. J Proteome Res.

[16] Knowles JR, Albery WJ (1977). Perfection in enzyme catalysis: the energetics of triosephosphate isomerase. Acc Chem Res.

[17] Seweryn K, Karkowska-Kuleta J, Wolak N, Bochenska O, Kedracka-Krok S, Kozik A, Rapala-Kozik M (2015). Kinetic and thermodynamic characterization of the interactions between the components of human plasma kinin-forming system and isolated and purified cell wall proteins of *Candida albicans*. Acta Biochim Pol.

[18] Peters BM, Jabra-Rizk MA, Scheper MA, Leid JG, Costerton JW, Shirtliff ME (2010). Microbial interactions and differential protein expression in *Staphylococcus aureus* -*Candida albicans* dual-species biofilms. FEMS Immunol Med Microbiol.

[19] Garcia-Sanchez S, Aubert S, Iraqui I, Janbon G, Ghigo JM, d’Enfert C (2004). *Candida albicans* biofilms: a developmental state associated with specific and stable gene expression patterns. Eukaryot Cell.

[20] Fernandez-Arenas E, Cabezón V, Bermejo C, Arroyo J, Nombela C, Diez-Orejas R, Gil C (2007). Integrated proteomics and genomics strategies bring new insight into *Candida albicans* response upon macrophage interaction. Mol Cell Proteomics.

[21] Kim KY, Shin YK, Kang KC, Yoo JS, Kim JH, Paik YK (2009). Proteomic profiling of yeast- and hyphal-specific responses of *Candida albicans* to the antifungal agent, HWY-289. Proteomics Clin Appl.

[22] Pitarch A, Abian J, Carrascal M, Sánchez M, Nombela C, Gil C (2004). Proteomics-based identification of novel *Candida albicans* antigens for diagnosis of systemic candidiasis in patients with underlying hematological malignancies. Proteomics.

[23] Pitarch A, Díez-Orejas R, Molero G, Pardo M, Sánchez M, Gil C, Nombela C (2001). Analysis of the serologic response to systemic *Candida albicans* infection in a murine model. Proteomics.

[24] Martinez-Lopez R, Nombela C, Diez-Orejas R, Monteoliva L, Gil C (2008). Immunoproteomic analysis of the protective response obtained from vaccination with *Candida albicans* ecm33 cell wall mutant in mice. Proteomics.

[25] Caminero A, Calvo E, Valentín E, Ruiz-Herrera J, López JA, Sentandreu R (2014). Identification of *Candida albicans* wall mannoproteins covalently linked by disulphide and/or alkali-sensitive bridges. Yeast.

[26] Vialás V, Perumal P, Gutierrez D, Ximénez-Embún P, Nombela C, Gil C, Chaffin WL (2012). Cell surface shaving of *Candida albicans* biofilms, hyphae, and yeast form cells. Proteomics.

[27] Rapala-Kozik M, Karkowska-Kuleta J, Ryzanowska A, Golda A, Barbasz A, Faussner A, Kozik A (2010). Degradation of human kininogens with the release of kinin peptides by extracellular proteinases of *Candida* spp. Biol Chem.

[28] Hoyer LL, S. Scherer S, A. R. Shatzman AR, Livi GP. *Candida albicans* ALS1: domains related to a *Saccharomyces cerevisiae* sexual agglutinin separated by a repeating motif. Mol Microbiol. 1995;15:39–54. 10.1111/j.1365-2958.1995.tb02219.x 7752895

[29] Kusch H, Engelmann S, Bode R, Albrecht D, Morschhäuser J, Hecker M (2008). A proteomic view of *Candida albicans* yeast cell metabolism in exponential and stationary growth phases. Int J Med Microbiol.

[30] Karkowska-Kuleta J, Wronowska E, Satala D, Zawrotniak M, Bras G, Kozik A, Nobbs A, Rapala-Kozik M (2020). The Als3-mediated attachment of enolase on the surface of *Candida albicans* cells regulates their interactions with host proteins. Cell Microbiol.

[31] Kinnby B, Booth NA, Svensäter G (2008). Plasminogen binding by oral streptococci from dental plaque and inflammatory lesions. Microbiol.

[32] Miranda-Ozuna JF, Hernández-García MS, Brieba LG, Benítez-Cardoza CG, Ortega-López J, González-Robles A, Arroyo R (2016). The glycolytic enzyme triosephosphate isomerase of *Trichomonas vaginalis* is a surface-associated protein induced by glucose that functions as a laminin- and fibronectin-binding protein. Infect Immun.

[33] Longo LVG, de Cunha JPC, Sobreira TJP, Puccia R (2014). Proteome of cell wall-extracts from pathogenic *Paracoccidioides brasiliensis*: Comparison among morphological phases, isolates, and reported fungal extracellular vesicle proteins. EuPA Open Proteomics.

[34] Harn DA, Gu W, Oligino LD, Mitsuyama M, Gebremichael A, Richter D (1992). A protective monoclonal antibody specifically recognizes and alters the catalytic activity of schistosome triose-phosphate isomerase. J Immunol.

[35] Braschi S, Curwen RS, Ashton PD, Verjovski-Almeida S, Wilson A (2006). The tegument surface membranes of the human blood parasite *Schistosoma mansoni*: a proteomic analysis after differential extraction. Proteomics.

[36] Jimenez-Sandoval P, Castro-Torres E, González-González R, Díaz-Quezada C, Gurrola M, Camacho-Manriquez LD, Leyva-Navarro L, Brieba LG (2020). Crystal structures of triosephosphate isomerases from *Taenia solium* and *Schistosoma mansoni* provide insights for vaccine rationale and drug design against helminth parasites. PLoS Negl Trop Dis.

[37] Furuya H, Ikeda R (2009). Interaction of triosephosphate isomerase from the cell surface of *Staphylococcus aureus* and α-(1→3)-mannooligosaccharides derived from glucuronoxylomannan of *Cryptococcus neoformans*. Microbiol..

[38] de Oliveira HC, Assato PA, Marcos CM (2015). Paracoccidioides-host Interaction: An overview on recent advances in the *Paracoccidioidomycosis*. Front Microbiol.

[39] Pereira LA, Báo SN, Barbosa MS, da Silva JLM, Felipe MS, de Santana JM, Mendes-Giannini MJ, de Almeida Soares CM (2007). Analysis of the *Paracoccidioides brasiliensis* triosephosphate isomerase suggests the potential for adhesin function. FEMS Yeast Research.

[40] Vargas G, Rocha JD, Oliveira DL (2015). Compositional and immunobiological analyses of extracellular vesicles released by *Candida albicans*. Cell Microbiol.

[41] Karkowska-Kuleta J, Kulig K, Karnas E (2020). Characteristics of Extracellular Vesicles Released by the Pathogenic Yeast-Like Fungi *Candida glabrata*, *Candida parapsilosis* and *Candida tropicalis*. Cells.

[42] Schwarz-Linek U, Höök M, Potts JR (2004). The molecular basis of fibronectin-mediated bacterial adherence to host cells. Mol Microbiol.

[43] Singh B, Fleury C, Jalalvand F, Riesbeck K (2012). Human pathogens utilize host extracellular matrix proteins laminin and collagen for adhesion and invasion of the host. FEMS Microbiol Rev.

[44] Piña-Vázquez C, Reyes-López M, Ortíz-Estrada G, de la Garza M, Serrano-Luna J (2012). Host-parasite interaction: parasite-derived and -induced proteases that degrade human extracellular matrix. J Parasitol Res.

[45] Kozik A, Karkowska-Kuleta J, Zajac D, Bochenska O, Kedracka-Krok S, Jankowska U, Rapala-Kozik M (2015). Fibronectin-, vitronectin- and laminin-binding proteins at the cell walls of *Candida parapsilosis* and *Candida tropicalis* pathogenic yeasts. BMC Microbiol.

[46] Frantz Ch, Stewart KM, Weaver VM (2010). The extracellular matrix at a glance. J Cell Science.

[47] Walker C, Mojares E (2018). Del Río Hernández A. Role of extracellular matrix in development and cancer progression. Int J Mol Sci.

[48] Hastings JF, Skhinas JN, Fey D, Croucher DR, Cox TR (2019). The extracellular matrix as a key regulator of intracellular signalling networks. Br J Pharmacol.

[49] Halper J, Kjaer M (2014). Basic components of connective tissues and extracellular matrix: elastin, fibrillin, fibulins, fibrinogen, fibronectin, laminin, tenascins and thrombospondins. Adv Exp Med Biol.

[50] Mouw JK, Ou G, Weaver VM (2014). Extracellular matrix assembly: a multiscale deconstruction. Nat Rev Mol Cell Biol.

[51] Leavesley DI, Kashyap AS, Croll T, Sivaramakrishnan M, Shokoohmand A, Hollier BG (2013). Vitronectin–master controller or micromanager?. IUBMB Life.

[52] Klotz SA (1994). Plasma and extracellular matrix proteins mediate in the fate of *Candida albicans* in the human host. Med Hypotheses.

[53] Gozalbo D, Gil-Navarro I, Azorín I, Renau-Piqueras J, Martínez JP, Gil ML (1998). The cell wall-associated glyceraldehyde-3-phosphate dehydrogenase of *Candida albicans* is also a fibronectin and laminin binding protein. Infect Imm.

[54] Gaur NK, Klotz SA, Henderson RL (1999). Overexpression of the *Candida albicans ALA1* gene in *Saccharomyces cerevisiae* results in aggregation following attachment of yeast cells to extracellular matrix proteins, adherence properties similar to those of *Candida albicans*. Infect Immun.

[55] Rauceo JM, de Armond R, Otoo H, Kahn PC, Klotz SA, Gaur NK, Lipke PN (2006). Threonine-rich repeats increase fibronectin binding in the *Candida albicans* adhesin Als5p. Eukaryot Cell.

[56] Klotz SA, Pendrak ML, Hein RC (2001). Antibodies to α5β1 and αvβ3 integrins react with *Candida albicans* alcohol dehydrogenase. Microbiol.

[57] Donohue DS, Ielasi FS, Goossens KV, Willaert RG (2011). The N-terminal part of Als1 protein from *Candida albicans* specifically binds fucose-containing glycans. Mol Microbiol.

[58] Jordan RPC, Williams DW, Moran GP, Coleman DC, Sullivan DJ (2014). Comparative adherence of *Candida albicans* and *Candida dubliniensis* to human buccal epithelial cells and extracellular matrix. Proteins Med Mycol.

[59] Satala D, Satala G, Karkowska-Kuleta J, Bukowski M, Kluza A, Rapala-Kozik M, Kozik A (2020). Structural insights into the interactions of candidal enolase with human vitronectin, fibronectin and plasminogen. Int J Mol Sci.

[60] Zajac D, Karkowska-Kuleta J, Bochenska O, Rapala-Kozik M, Kozik A (2016). Interaction of human fibronectin with *Candida glabrata* epithelial adhesin 6 (Epa6). Acta Biochim Pol.

[61] Ielasi FS, Decanniere K, Willaert RG (2012). The epithelial adhesin 1 (Epa1p) from the human pathogenic yeast *Candida glabrata*: structural and functional study of the carbohydrate-binding domain. Acta Crystallogr D Biol Crystallogr.

[62] Rapala-Kozik M, Karkowska J, Jacher A, Golda A, Barbasz A, Guevara-Lora I, Kozik A (2008). Kininogen adsorption to the cell surface of *Candida* spp. Int Immunopharmacol.

[63] Matsudaira P (1987). Sequence from picomole quantities of proteins electroblotted onto polyvinylidene difluoride membranes. J Biol Chem.

[64] Abramoff MD, Magalhaes PJ, Ram SJ (2004). Image Processing with ImageJ. Biophotonics International.

[65] Xu H, Freitas MA (2009). MassMatrix: A database search program for rapid characterization of proteins and peptides from tandem mass spectrometry data. Proteomics.

[66] Sali A, Blundell TL (1993). Comparative protein modelling by satisfaction of spatial restraints. J Mol Biol.

[67] Vajda S, Yueh C, Beglov D, Bohnuud T, Mottarella SE, Xia B, Hall DR, Kozakov D. New additions to the ClusPro server motivated by CAPRI. Proteins. 2017;85:435–444. 10.1002/prot.25219 PMC5313348 27936493

[68] Kozakov D, Hall DR, Xia B, Porter KA, Padhorny D, Yueh C, Beglov D, Vajda S (2017). The ClusPro web server for protein-protein docking. Nature Protocols.

