# Supplementary material for: Candida albicans and Candida glabrata triosephosphate isomerase – a moonlighting protein that can be exposed on the candidal cell surface and bind to human extracellular matrix proteins
Source: BMC Microbiol. 2021 Jul 1;21:199. doi: 10.1186/s12866-021-02235-w (PMC8252264; doi:10.1186/s12866-021-02235-w)
Supplement: Supplementary file 2 — Additional file 1: Table S1. List of peptide matches obtained with the Mass Matrix server. Figure S1. The analysis of cross-reactivities of anti-S. cerevisiae Tpi1 antibody to C. albicans and C. glabrata Tpi1. Figure S2. Identification of Tpi1 on the surface of C. albicans and C. glabrata cells, grown in YPD and YPDA medium. Figure S3. SDS-PAGE analysis of purified Tpi1 from C. albicans and C. glabrata. Figure S4. SPR sensograms for the interactions of C. albicans and C. glabrata Tpi1 with human ECM proteins: FN, LAM, COL and ELA. Figure S5. Relative enzymatic activity of C. albicans and C. glabrata Tpi1 after interaction with human ECM proteins. [file 12866_2021_2235_MOESM1_ESM.pdf]

Supplementary Material

Candida albicans and *Candida glabrata* triosephosphate isomerase—a moonlighting protein that can be exposed on the candidal cell surface and bind to human extracellular matrix proteins

Dorota Satala[1], Grzegorz Satala[2], Marcin Zawrotniak[3], Andrzej Kozik[1*]

[1]Department of Analytical Biochemistry, Faculty of Biochemistry, Biophysics and Biotechnology, Jagiellonian University in Krakow, Kraków, Poland

[2]Department of Medicinal Chemistry, Maj Institute of Pharmacology, Polish Academy of Sciences, Kraków, Poland

[3]Department of Comparative Biochemistry and Bioanalytics, Faculty of Biochemistry, Biophysics and Biotechnology, Jagiellonian University in Krakow, Kraków, Poland

*corresponding author: [1]Department of Analytical Biochemistry, Faculty of Biochemistry, Biophysics and Biotechnology, Jagiellonian University in Krakow; Gronostajowa 7, 30-384 Krakow, Poland; e-mail: andrzej.kozik@uj.edu.pl

Table S1. List of peptide matches obtained with the Mass Matrix server.

In the sequences identified, amino acids were underlined for which mass shifts due to the sulfo-SDAD-derived link are indicated.

Peptide	Charge	Score	pp value	pp2 value
$_{4}$QFFVGGNFKANGTK$_{17}$	+3	46.4	10.3	7.6
$_{224}$ANVDGFLVGGASLKPEFVDIIKSR$_{247}$	+3	70.4	16.3	14.1

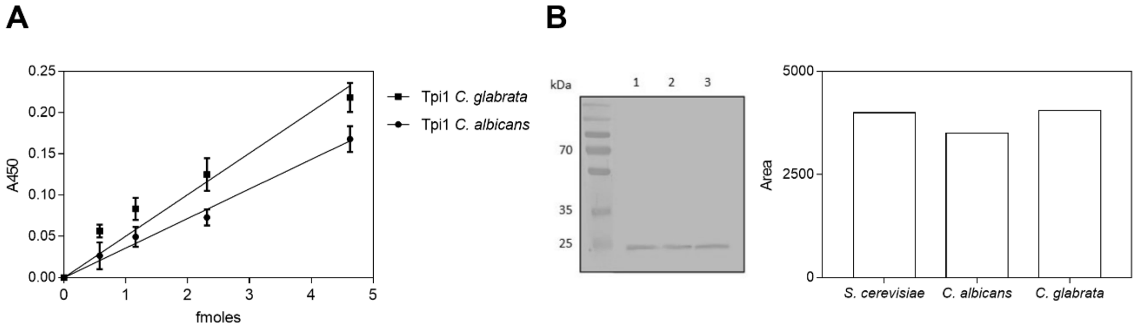

Figure S1. The analysis of cross-reactivities of anti-*S. cerevisiae* Tpi1 antibody to *C. albicans* and *C. glabrata* Tpi1.

(A) A calibration curve for microplate assays. Microplate wells were coated with Tpi1 at amounts in a range of 0.5–4.5 fmoles (50 µL of protein solution in PBS) in an overnight incubation at 4˚C. After each step of the following assay, the wells were washed three times with 200 µL PBS, pH 7.4, containing 1% BSA. The unoccupied microplate well surface was blocked by incubation with 200 µL of 3% BSA in PBS for 1 h at 37°C. After addition of 50 µL of anti-yeast Tpi1 antibody (1 µg/mL), the plates were incubated for 1 h at 37°C. Next, 50 µL of HRP-conjugated secondary antibody were added, for further plate incubation at 37°C for 1 h and color development with SA-HRP/TMB system.

(B) A calibration of western-blotting experiments. Equal amounts (0.3 µg) of purified Tpi1 were applied to the gel wells and separated by SDS-PAGE in the Laemmli system under reducing conditions. The protein bands were then transferred to a PVDF membrane and probed with primary anti-*S. cerevisiae* Tpi1 antibodies and an alkaline phosphatase-labelled secondary antibody. Protein bands were visualized using a BCIP/NBT system. The uncropped scan of the blot is presented (panel left). Lane 1, 0.3 µg of *S. cerevisiae* Tpi used as a control; lane 2, 0.3 µg of *C. albicans* Tpi1; lane 3, 0.3 µg of *C. glabrata* Tpi1. The amount of Tpi1 recognized by anti-*S. cerevisiae* Tpi antibodies were determined by densitometric analysis

using ImageJ software (panel right). The obtained results were used to normalize the results with the use of anti-*S. cerevisiae* Tpi antibodies.

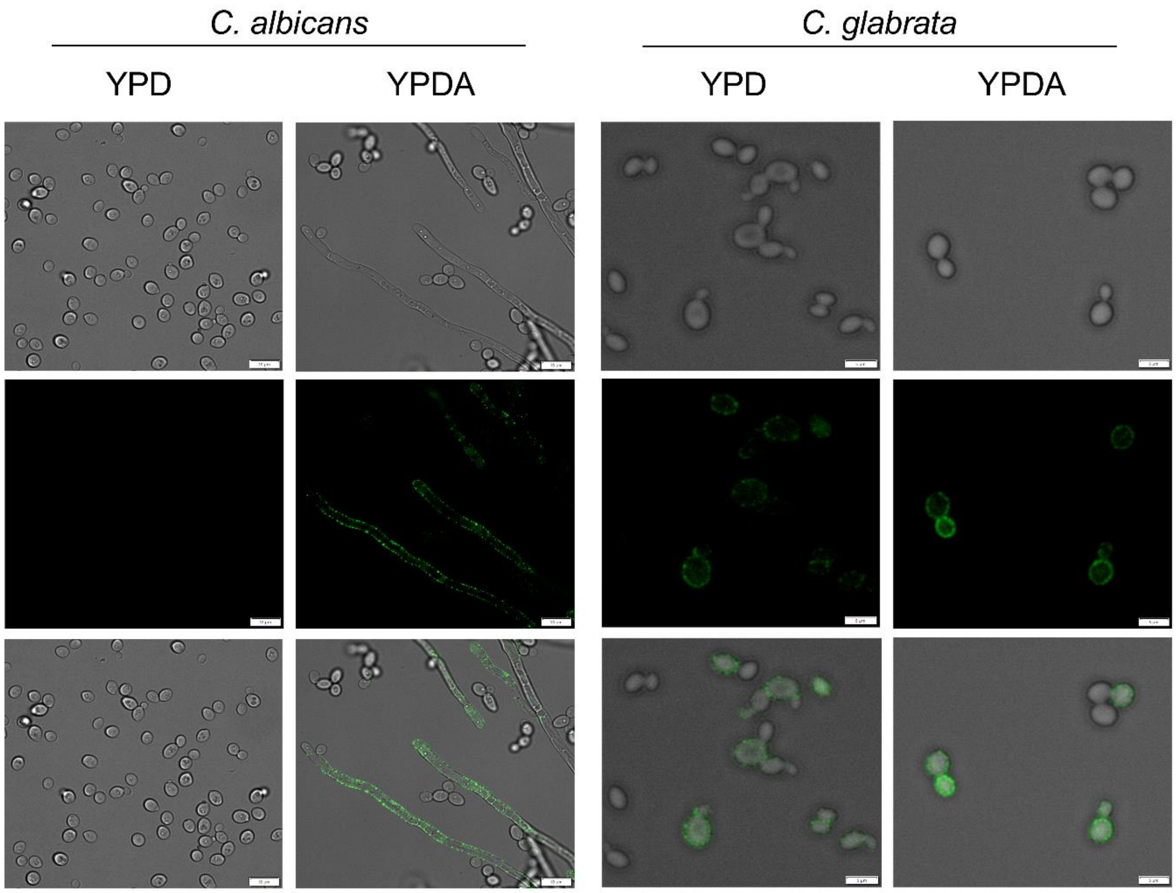

Fig. S2. Identification of Tpi1 on the surface of *C. albicans* and *C. glabrata* cells, grown in YPD and YPDA medium.

C. albicans yeast-like cells, grown for 16 h in YPD medium at 30°C and then transferred to Eppendorf tubes (3 x 10^7 cells per tube) or transferred to the wells of a glass-bottom 96-well plate coated with poly-L-lysine (10^4 cells per well) and incubated in YPDA medium at 37°C overnight. *C. glabrata* yeast-like cells were grown overnight in YPD or YPDA medium in a glass flask and then transferred to Eppendorf tubes (3 x 10^7 cells per tube). The presence of surface-exposed Tpi1 was detected using rabbit polyclonal anti-Tpi antibody (1 μg/mL) and an Alexa Fluor 488 fluorescently labeled anti-rabbit secondary antibody (1 μg/mL). Photo of bright field (top row) and green fluorescence (middle row) were taken using an Olympus IX73 microscope. The bottom row shows the overlapped images.

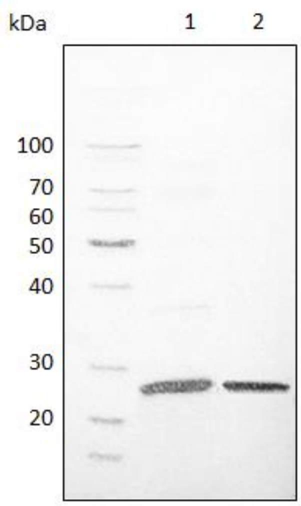

Figure S3. SDS-PAGE analysis of purified Tpi1 from *C. albicans* and *C. glabrata*.

Purified Tpi1 samples from *C. albicans* and *C. glabrata* were analysed with the Laemmli system using a 12% gel under reducing conditions. After electrophoresis, the protein bands were visualized by silver staining. The uncropped scan of the gel is presented. Molecular markers are shown in the leftmost lane. Lane 1, purified *C. albicans* Tpi1; lane 2, purified *C. glabrata* Tpi1.

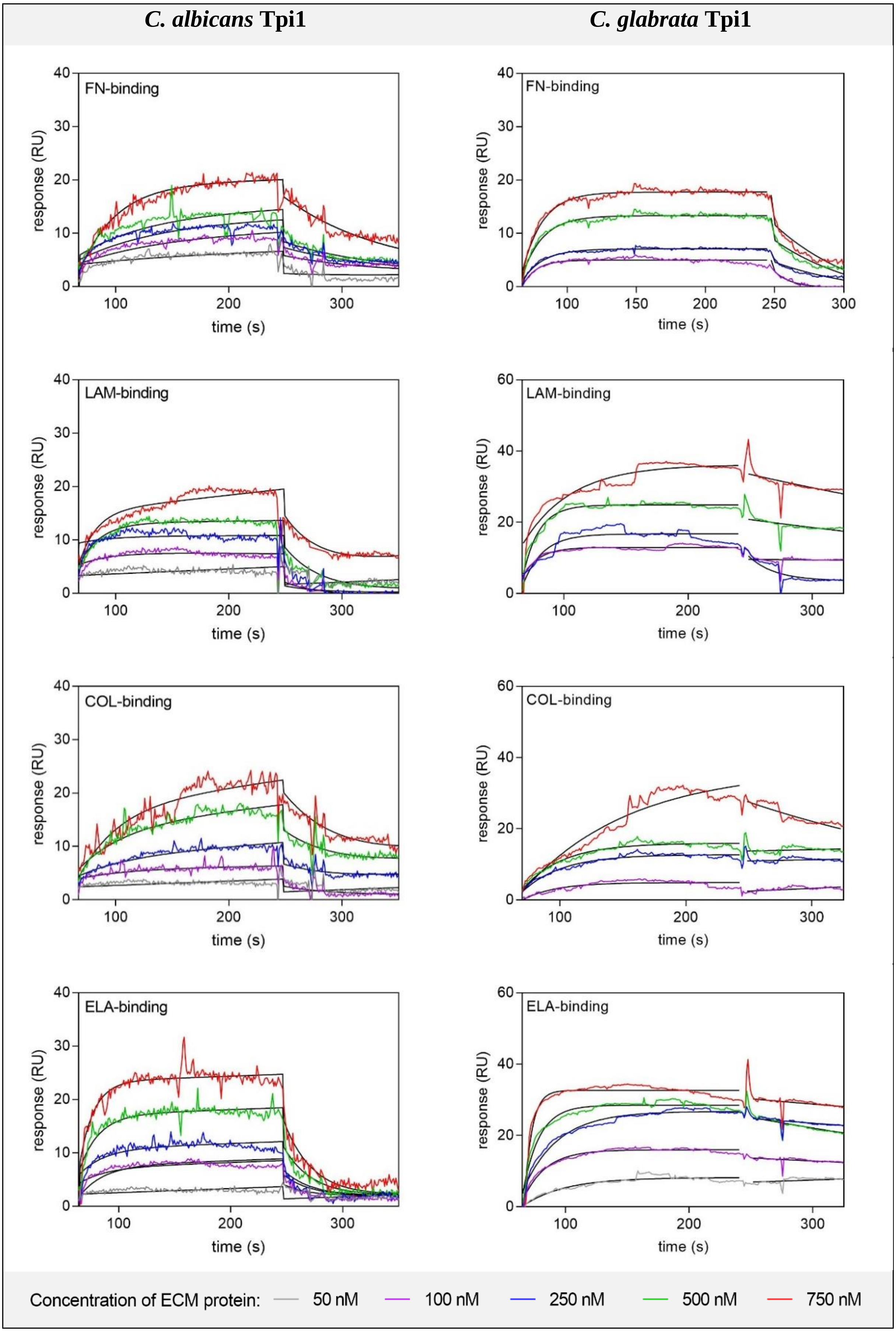
C. albicans Tpi1
C. glabrata Tpi1
FN-binding
LAM-binding
COL-binding
ELA-binding
response (RU)
time (s)
Concentration of ECM protein:
50 nM
100 nM
250 nM
500 nM
750 nM

Figure S4. SPR sensograms for the interactions of *C. albicans* and *C. glabrata* Tpi1 with human ECM proteins: FN, LAM, COL and ELA.

Human protein solutions in the buffer containing 10 mM HEPES, 150 mM NaCl and 0.005% (w/v) P20 surfactant, pH 7.4, at a concentration range of 50-750 nM, were injected onto a CM5 chip containing immobilized Tpi1 (150 RU) at a flow rate of 30 μL/min for 180 seconds. A 1:1 Langmuir binding model was fitted to the experimental data (black lines).

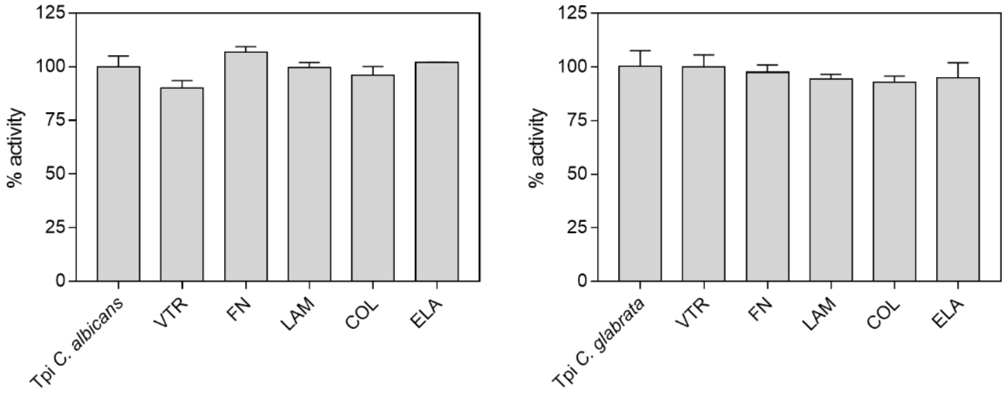

Figure S5. Relative enzymatic activity of *C. albicans* and *C. glabrata* Tpi1 after interaction with human ECM proteins.

Tpi1 was incubated with ECM proteins at a 1:1 molar ratio for 1 hour in PBS at 37°C, with gentle shaking. The results show the values obtained after 40 min of activity monitoring and are presented as a percentage of the control experiment ("no human protein") taken as 100%. The bars represent mean values ± standard deviations (3 determinations).
